# Diagnosis and treatment of early-stage endometriosis by Transvaginal Hydro laparoscopy

**DOI:** 10.52054/FVVO.15.1.057

**Published:** 2023-03-31

**Authors:** S Gordts, P Puttemans, I Segaert, M Valkenburg, V Schutyser, R Campo, Sy Gordts

**Affiliations:** Leuven Institute for Fertility & Embryology, Schipvaartstraat 4, 3000 Leuven, Belgium; Life Expert Centre, Schipvaartstraat 4, 3000 Leuven, Belgium; Hospital Heilig Hart, Naamsestraat 105, 3000 Leuven, Belgium.

**Keywords:** Infertility, endometriosis, transvaginal hydro laparoscopy, diagnosis, treatment, pregnancy

## Abstract

**Background:**

Transvaginal Hydro Laparoscopy (THL) is known as a minimal invasive procedure allowing endoscopic exploration of the female pelvis.

**Objective:**

To evaluate the possibilities of the THL as a tool for early diagnosis and treatment of minimal endometriosis.

**Materials and methods:**

A retrospective study of a consecutive series of 2288 patients referred for fertility problems to a tertiary centre for reproductive medicine was undertaken. Mean duration of infertility was 23.6 months (SD ±11-48), mean age of patients was 31.25 (SD± 3.8y). With normal findings at clinical and ultrasound examination patients underwent, as part of their fertility exploration, a THL.

**Main outcome measures:**

Evaluation of feasibility, identified pathology and pregnancy rate.

**Results:**

Endometriosis was diagnosed in 365 patients (16%); the localisation was higher on the left side (n=237) than on the right side (n=169). Small endometriomas, with diameters between 0.5 and 2 cm, were present in 24.3% (right side in 31, left side 48 and bilateral 10). These early lesions were characterised by the presence of active endometrial like cells and a pronounced neo-angiogenesis. Destruction of the endometriotic lesions with bipolar energy resulted in an in vivo pregnancy rate (spontaneous/IUI) of 43.8% (CPR after 8 months: spontaneous 57.7%; IUI/AID 29.7%).

**Conclusion:**

THL allowed in a minimally invasive way an accurate diagnosis of the early stages of peritoneal and ovarian endometriosis with the possibility of offering treatment with minimal damage.

**What is new?:**

This is the largest series reporting the usefulness of THL for the diagnosis and treatment of peritoneal and ovarian endometriosis in patients without obviously visible preoperative pelvic pathology.

## Introduction

With an estimated incidence of 2% – 22 % in the general population, the incidence of endometriosis, diagnosed at laparoscopy in the infertile female is reported to range between 20% - 50% (Moen, 1987). Although the causal relationship between endometriosis and infertility, in the absence of adhesions, has not clearly been proven up till now, it is generally accepted upon epidemiological data, that endometriotic implants are responsible for diminished fertility. Treatment of these lesions remains debatable if there is no proven causal relationship.

In a recent retrospective study of patients with so called “unexplained infertility” it was found that when these patients had untreated minimal or mild endometriosis the time to natural conception was significantly prolonged in comparison to patients without lesions (Akande et al., 2004; Duffy et al., 2014). This together with the results of the Canadian Collaborative study Group on ablation of minimal and mild endometriosis (Marcoux et al., 1997), support the view that detecting and treating these lesions is beneficial in sub-fertile women. Although endometriosis can have a huge negative impact on the quality of life due to chronic pelvic pain, dysmenorrhoea, deep dyspareunia and dyschezia, diagnosis is systematically delayed even when symptoms start early in adolescence (Arruda et al., 2003; Nnoaham et al., 2011; Hadfield et al., 1996; Dmowski et al., 1997). In adolescents with chronic pelvic pain, the incidence of endometriosis has been reported between 9% and 73% (Davis et al., 1993; Reese et al., 1996; Laufer et al., 2003). An increase in the severity of endometriosis has been reported during the last few decades with an estimated incidence of severe endometriosis stage III –IV before 2000 of 11% and 59% in papers published after 2000 (Brosens et al., 2013). As a linear decline in fertility with each stage of endometriosis was described (Ventolini et al., 2005), early diagnosis is considered important to preserve adolescents’ fertility potential. The presence of ovarian endometriomas is a burden, as the disease itself causes diminished ovarian reserve. Cystic ovarian endometriosis is strongly associated with pelvic adhesions, which can obviously cause infertility because of a disturbed tubo-ovarian relation with impaired ovum transport and pick-up mechanism. The underlying relationship between endometriosis and infertility could be a cause-and-effect relationship or both could have a common cause. The heterogeneity of the disease is so complex that it can hardly be explained by a simple mechanism. The mostly accepted pathophysiological explanations for the origin of endometriosis are the Sampson theory with retrograde menstruation and implantation (Sampson, 1927), and the metaplasia theory (Fukunaga, 2000). More recently the Genetic- Epigenetic theory has been suggested as a possible explanation. It is based upon the assumption that a specific genetic-epigenetic alteration will induce cellular alterations creating a predisposition for the development of endometriosis. As such the infertility will not be caused by the endometriotic lesion itself but by the “endometriotic constitution” (Koninckx et al., 2021). The endometriotic lesions are the consequences of this constitution, a theory which has also been suggested for adenomyosis. In this optic it could be of interest to treat the endometriosis at an early stage avoiding progression to more severe forms, particularly in the case of ovarian cysts results wherein pronounced fibrosis develops and diminished ovarian reserve (Kitajima et al., 2014). Treatment performed in these early stages has the potential to cause minimal trauma and less adhesion formation. The delay in the diagnosis of endometriosis of 3-11 years (Arruda et al., 2003) between the initial onset of symptoms and the final diagnosis is a burden and unacceptable certainly in the young patients. We report on the use of transvaginal hydro laparoscopy (THL) as a minimally invasive procedure in the early diagnosis and treatment of peritoneal and ovarian endometriosis up to a diameter of 2 cm in a consecutive series of 2288 diagnostic THL procedures.

## Materials and methods

In a recent publication we described our findings of a retrospective analysis of 2288 consecutive diagnostic transvaginal hydro-laparoscopies performed as part of the fertility exploration in couples with primary or secondary infertility and without obvious pelvic pathology (Gordts et al., 2021). As previously mentioned, contra-indications for the THL were obliterated pouch of Douglas due to a fixed retroverted uterus or presence of deep infiltrating endometriosis (DIE) and presence of ovarian endometrioma larger than 2 cm. Its use is restricted to those patients without obvious pelvic pathology: normal clinical examination and normal ultrasound. We described the THL technique in previous publications (Campo et al., 1999; Gordts et al., 1998). Briefly, access to the pelvis is gained by a simple needle puncture of the pouch of Douglas. The pelvis is accessed with a reusable, spring-loaded needle with an adjustable length (1.0 cm to 2.5 cm), which proved to be advantageous dealing with obese patients. The advantage of the spring-loaded needle is the speed once the needle is shut, avoiding tenting of the peritoneum, and minimising the risk of access failure. Patients were placed under conscious sedation, in a gynaecological position, without Trendelenburg. All procedures were performed in a one-day hospital setting.

As at THL, the longitudinal axis of the endoscope is parallel to the tubo-ovarian axis, positioning the scope during THL provide direct access to the tubo-ovarian structures and the ovarian fossa, without extra manipulation. It allows the inspection of the lesions with the organs in a physiological status, in contrast to standard laparoscopy where visualisation of the fossa and the anterior site of the ovary, requires grasping and turning of the ovarian ligament, frequently resulting in rupture of adhesions and possible bleeding. The use of a watery distension medium increases the accuracy for detection of adhesions (Campo et al., 1999) compared to the use of CO_2_; the latter causing a high intra- abdominal pressure with a possibility of masking filmy adhesions.

**Figure 1:**
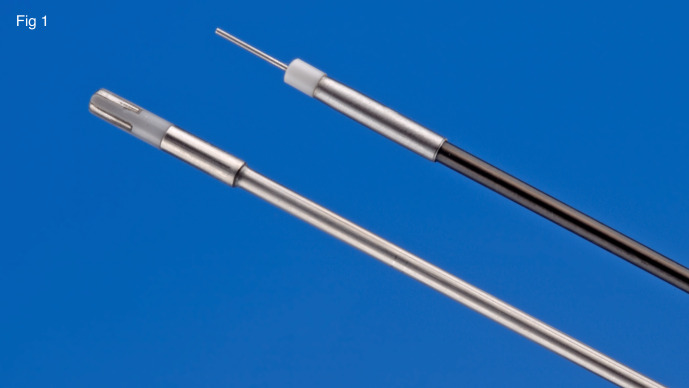
Bipolar needle and coagulation probe.

Working under water requires the possibility of a perfect haemostasis and following the microsurgical principles vessels should first be coagulated and then cut to avoid any uncontrolled bleeding. Mandatory with the use of a watery distension medium is the use of bipolar current. Two 5 Fr. bipolar instruments are used for this purpose: a 0.2 mm bipolar needle for cutting or punctiform coagulation and a 5Fr bipolar coagulation probe (Bicap°) (Karl Storz, Germany) used for coagulation and for the ablative destruction of the pseudo-endometriotic cystic wall (Figure 1). Carbonisation, sticking of the tissue to the probe and undesirable deep coagulation are excluded and coagulation remains superficial. Endometriosis in these small ovarian cysts are superficial and as such a superficial destruction should be sufficient.

All patients provided informed consent before the procedure. In addition, patients were informed that, in case of complication, the operation could be converted to standard laparoscopy. All patients agreed that their data could be used anonymously for research. This retrospective study involved the collection of existing data recorded for procedures routinely performed in exploring the pelvis of infertile patients; therefore, institutional review board approval was not mandatory.

### Surgical procedure

In previous publications we described the different steps of the operative procedure when endometriosis is detected at THL (Gordts et al., 2014).

In the early stages of ovarian endometriosis, the ovarian fossa is a preferential place of implantation of active endometrial-like tissue and adhesion formation, which are almost always present. These adhesions can be filmy, non-connecting adhesions covering the invaginated brownish endometriotic spot, or can be more fibrotic, fixing the small ovarian endometrioma to the pelvic side wall. Small brownish or red spots or vesicles can hide a deeper infiltrated lesion only becoming visible after opening. What appears as a superficial vesicle on the ovarian surface is mostly an invaginated lesion filled with brownish or bloody content and covered by filmy translucent adhesions. Superficial ovarian and peritoneal vesicular lesions and adhesions were destroyed using a 5 Fr bipolar coagulation probe.

In case of fixed adhesions, first the normal anatomy is restored by adhesiolysis with 5Fr scissors. The likelihood of the presence of an endometrioma increases in the presence of fibrotic adhesions fixing the ovary with the pelvic sidewall. When the ovary was fixed with filmy or more fibrous adhesions in the fossa, endometrial-like tissue was identified at dissection. All cysts caused an invagination of the ovarian cortex; adhesions covered the site of invagination or fixed the ovary to the pelvis in the ovarian fossa.

**Figure 2:**
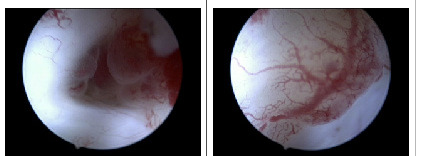
Ovarioscopy of small endometrioma of 1.5 cm showing presence of endometrial-like tissue and neo-angiogenesis.

The small endometrioma is opened using the bipolar needle and 5Fr scissors. After opening and its chocolate content drained, an ovarioscopy exploring the inside of the endometrioma is performed. The visual information obtained in this way was the same in all cases showing the inflammatory and aggressive character of the disease in these early stages: active red flame like implants, with a 3D aspect of cobble stones and marked neovascularisation towards the lesions on a pearl-white background (Figure 2). The endometriotic implants inside an endometrioma can easily be peeled off and sent to pathology. Implants and the surrounding neovascularisation were coagulated selectively under direct vision using a 5 Fr bipolar coagulation probe (Bicap°) (K. Storz, Germany) which resulted in a white surface without carbonisation and with minimal trauma.

## Results

**Figure 3:**
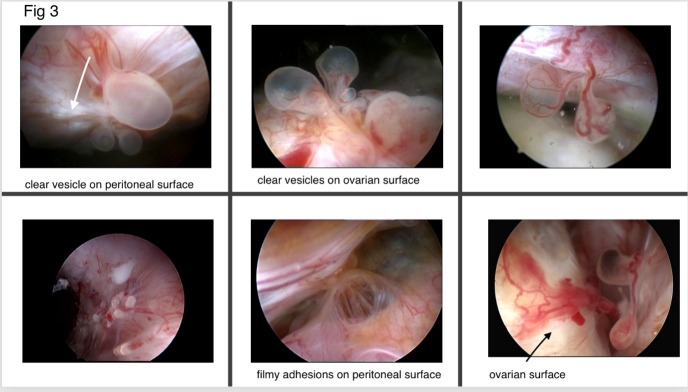
Presence of clear and red vesicles upon the peritoneal and ovarian surface; remark the presence of blood vessels at the base of the polyp and white area with beginning of fibrosis (white arrow).

In a consecutive series of 2288 THL as part of a routine fertility exploration in patients without obvious pelvic pathology, endometriotic lesions were diagnosed in 15.9 % (n=365) of patients with a mean duration of infertility of 26.5m (± 2.6). The incidence was higher on the left side (n=237) than on the right side (n=169). Lesions appeared as brown, red, or clear polypoid vesicles on the ovarian or peritoneal surface, frequently covered with filmy adhesions. At the base of the polyps small blood vessels are visualized and fibrotic areas can be present at the site of implantation of the polyps with at the surroundings signs of neo-angiogenesis (Figure 3). In total 99 small endometrioma with diameters between 0.5 and 2 cm were identified: 48 on the left side, 31 right and bilateral in 10 patients. After slicing open these small endometriomas and rinsing out the brown content, an ovarioscopy showed the presence of neo-angiogenesis, blebs of endometrial-like tissue, and in some cases, a gladder fibrotic wall. If a biopsy was obtained, histology showed the presence of small fibrotic tissue fragments with endometrial glands surrounded by endometrial stroma. It showed active endometriotic foci with presence of iron loaded macrophage. In nineteen patients. due to the presence of multiple adhesions or extensive endometriotic lesions, referral to a standard laparoscopy was required. Among these, nine patients refused laparoscopy. In the other ten patients, laparoscopy confirmed the THL findings of several peri-ovarian and peri-tubal adhesions, which were too extensive for THL treatment. The detection of these lesions at THL demonstrates the added value of the endoscopic exploration of the pelvis in these patients as findings at clinical examination and ultrasound were normal. Recurrence of endometriotic cyst occurred in five out of the 89 patients with treated ovarian endometrioma (5.6%). Because of a bleeding at the hilus due to the operative procedure of adhesiolysis, one patient was converted to standard laparoscopy to enable haemostasis and further treatment. No other complications occurred, and all patients could leave the hospital the same day. Postoperative control after four weeks was uneventful. The failure rates and complication rates of respectively 1% and 0.74% in our series are very low (Gordts S et al., 2021). Other publications report a failure and complication rate of 2.5% and 4.5% ((van Kessel et al., 2018; Verhoeven et al., 2004).

**Figure 4:**
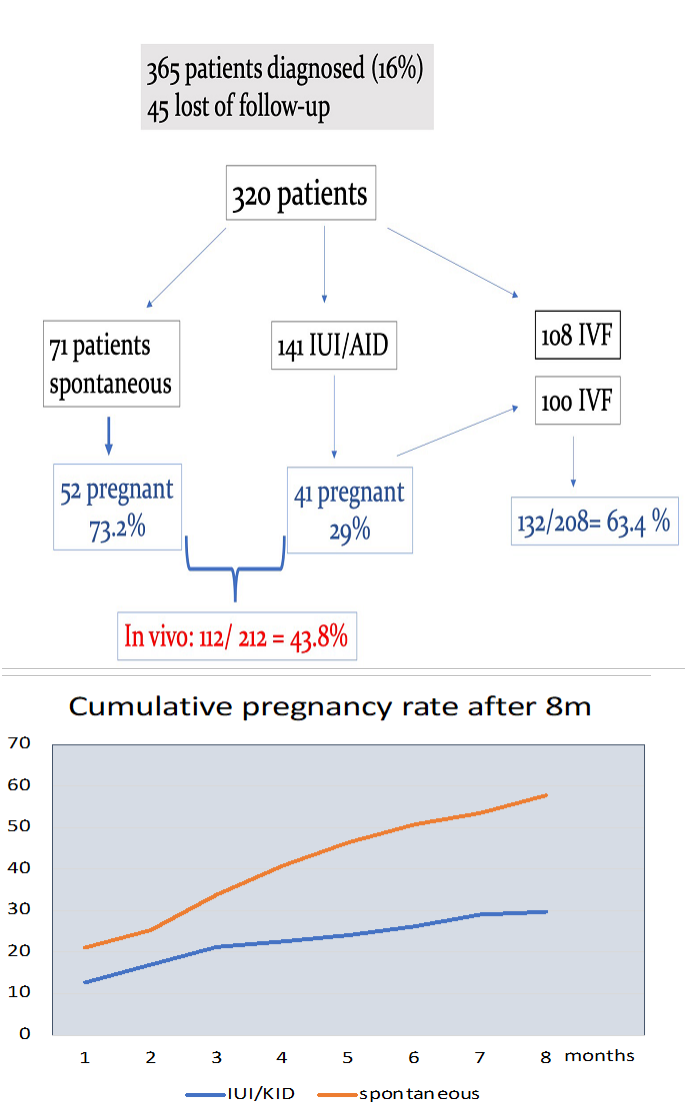
Results after treatment of peritoneal and ovarian endometriosis: in absence of other fertility impairing factors the spontaneous pregnancy rate was 73.2%.

Of the 365 patients with endometriotic lesions, 45 did not return for further treatment, and they were lost of follow-up. Of the remaining 320 patients and in absence of other fertility impairing factors, 71 patients were counselled to attempt spontaneous conception, and 52 achieved pregnancies (73.2%). The cumulative pregnancy rates after 3, 6, 12, and 24 months were, respectively, 33.8%, 50.7%, 63.3%, and 66.1%. Another 141 patients were treated with an intra uterine insemination (IUI) or artificial insemination with donor sperm (AID), resulting in 41 pregnancies (29%). The cumulative pregnancy rates after 3, 6, and 8 months in this group were, respectively, 21.2%, 26.2%, and 29%. In the combined group of spontaneous conception and IUI/KID (n= 212), the in vivo pregnancy rate was 43.8% (93/212). Of the 100 patients that did not achieve pregnancies with an IUI/AID treatment, 29 stopped the treatment, 5 are currently being treated, and 66 patients were referred to an IVF treatment, which resulted in 50 pregnancies (75.5%). Due to a combination of male subfertility, age, or a poor ovarian reserve and after discussion with the couple, 108 patients were directly referred to an IVF treatment, which resulted in 82 pregnancies (75.9%) (Figure 4).

## Discussion

Transvaginal ultrasound is generally considered as a useful method for early detection of the ovarian endometrioma and proposed as a reliable method to exclude significant ovarian endometriosis in patients with infertility (Brosens et al., 2004). However, a recent critical review on the accuracy of ultrasound in the diagnosis of endometriosis found that the published prospective studies all included endometriomas with a diameter of at least 14mm (Moore et al., 2002). Its accuracy in the diagnosis of endometriomas < 14mm is not known. Although MRI has been reported to be able to detect endometriotic lesions as small as 3mm (Takahashi, 1994) subtle peritoneal lesions and adhesions are not diagnosed using indirect methods of visualisation.

At laparoscopy subtle lesions and superficial adhesions are frequently missed as they are masked due to the high abdominal pressure of the CO2 pneumoperitoneum. The use of hydroflotation (Laufer, 1997) prevents the collapse of filmy adhesions and allows the visualisation of subtle lesions. THL has been described as a more sensitive technique than standard laparoscopy to detect subtle ovarian lesions (Campo et al., 1999).

The systematic omission of endoscopic visualisation of the pelvis in the exploration of the infertile patient results in undiagnosed lesions of endometriosis, tubal pathology and adhesions and an increased incidence of so called “unexplained infertility”. The cohort of patients referred for IVF in our centre with the diagnosis of unexplained infertility is 5.83% versus a mean of 21.63% in the other Belgian centres, as documented in the Belrap report of 2017 (Belrap, 2017). Pantou et al. (2019) reported the presence of endometriosis in 57.94% and 23.3% in a cohort of 107 patients with unexplained infertility referred for laparoscopy after three failed IVF attempts. Correcting these pathologies at laparoscopy resulted in a spontaneous cumulative pregnancy rate of 50-60 %. In a smaller study including 45 patients with unexplained infertility and failed IVF (Yu et al., 2019) the reported presence of endometriosis, tubal pathology and adhesions was respectively 57.7 %, 31.1% and 33.3% of the patients. In the study of Kanda et al. (2006) the diagnosis of unexplained infertility before and after laparoscopy decreased from 48.9 % to 19.5%. Laparoscopy revealed the presence of endometriosis (50.7%), tubal pathology (6.5%) and adhesions (2.8%) in the other patients. If diagnostic exploration of the infertile patient is limited to indirect imaging techniques early lesions of endometriosis and tubo-ovarian adhesions will be missed. In a recent publication (van Kessel et al. 2018) the cumulative spontaneous pregnancy rates were significantly reduced when endometriosis and/or adhesions were diagnosed at THL compared to normal findings. On the other hand, the impact of these lesions on the results after IVF treatments is less clear.

In our study we are evaluating the effect of treatment on the possibility of achieving a pregnancy as we have no conclusive data on dysmenorrhoea or chronic pelvic pain. The surgical ablative treatment of the early lesions of endometriosis can be discussed, but in absence of specific markers of progressivity, it is unclear if these lesions after a while will fade out or evolute to more severe forms of endometriosis. Early treatments of these lesions cause less ovarian trauma, compared to surgery on larger ovarian cyst. The magnitude of the ovarian damage might be related to the size of cyst and the damage to ovaries is more severe when an endometrioma >4 cm is excised (Tang et al., 2013). A direct proportional relationship between endometrioma size and inadvertent removal of ovarian parenchyma has been described (Roman et al., 2010). In contrast to the laparoscopic operative procedures for endometriosis where the debate is still going on between cystectomy or ablative surgery, this discussion is not of interest in the treatment of these small endometriotic cysts where only an ablative approach is possible. We can hypothesise that in these early lesions the fibrotic reaction, as seen in more severe forms of endometriosis, still is minimal and as there is practically no distension of the cystic wall, cystectomy caries an increased risk of inadvertent removal of primordial follicles with a greater injury to the ovarian reserve (Romualdi et al., 2011). Recent data showed less destruction using an ablative technique compared to the cystectomy with same risks of recurrences and less ovarian trauma as expressed by AMH and antral follicle count (Candiani et al., 2018; Roman et al., 2011).

In contrast to the more severe forms of endometriosis where there is frequently absence of endometrial like tissue insight the ovarian cyst (Muzii et al., 2007), in these early stages endometrial like tissue and neo-angiogenesis were prominently identified in all cysts.

In THL the whole procedure is performed under water therefore minimising the risk of adhesion formation. As watery distension medium a Ringer lactate solution is used as this showed beneficial in the prevention of adhesion formation compared with a saline solution (Elkelani et al., 2002). A recent study performing second look THL in patients receiving a standard laparoscopic drilling versus an ovarian drilling done by THL showed the presence of adhesion formation in 70.2 % of the patients if drilling was done by standard laparoscopy versus only 15.5 % when done by THL (p<0.0001) (Giampaolino et al., 2016). Adding to the minimised risk of adhesion formation, is the use of the bipolar coagulation probe (Bicap°) where the depth of coagulation is superficial. The possibility of this probe rinsing when coagulating, reduces heating and avoids carbonisation. As endometriosis in cystic ovarian endometrioma is superficial and certainly in these early stages, a superficial destruction should be sufficient.

The watery distension medium allows for accurate visualisation of the impressive signs of neovascularisation and small vesicles at peritoneal and ovarian level, frequently masked at standard laparoscopy due to the use of the pneumoperitoneum. In these red, brown and clear vesicular lesions endometriosis was confirmed in 75-97% of the examined biopsies (Donnez et al., 1994; Martin et al., 1989; Stripling et al., 1988). Although not all lesions have been biopsied in our patients, histological examination of biopsies of these early lesions showed presence of small fibrotic tissue fragments including endometrial glands surrounded by endometrial stroma with presence of iron loaded macrophages compatible with an active endometriosis hearth. Mostly fibrotic tissue fragments showed presence of endometrial stroma and endometrial glands. Because of the close inspection under water at THL in these early stages of endometriosis at dissection of these adhesions less fibrosis is present and mostly active endometrial like tissue can be identified, showing at some places invasion into the parietal wall. We believe that the treatments in these early stages of endometriosis can avoid further fibrosis of these adhesions and will diminish fibrotic intra-ovarian reaction harming the ovarian reserve (Kitajima et al., 2014). As described previously endometriotic lesions were more frequent on the left site than right. This finding is also confirmed in these early stages of development of endometriosis indicating on the possible role of the anatomical created space with the presence of the sigmoid compared to the right. Although progression of the disease is difficult to predict, aggressiveness of the lesions even in small cyst <2cm should not be underestimated as shown at intra cystic exploration by the presence of this pronounced neo-angiogenesis and inflammatory reaction. If the risk of initiating endometriosis is the highest at younger age (Koninckx et al., 2021) and decreases progressively thereafter, an early diagnosis and treatment should be considered.

The added effect of THL in the exploration of the infertile patient is demonstrated by the fact that 19 patients were referred for a standard operative laparoscopic procedure as the lesions were too extensive to be treated by THL. These lesions were not detected at clinical or ultrasound examination just before the start of the THL procedure, as routinely an ultrasound examination is performed in the operating room.

The low prevalence of 15.9% of patients with endometriosis in our consecutive series of 2288 THL can be explained by the fact that all these patients had a normal clinical examination and normal ultrasound; the incidence in our study is comparable by the hazardous finding of endometriosis in 21.8% in sub-fertile patients referred for IVF treatment with the presence of an ovarian endometrioma in 10.5% of the patients (Alson et al., 2022). If endometriosis pathology was diagnosed by ultrasound or clinical examination these patients are not included in our study as they were referred for a standard operative laparoscopic procedure.

We must stress the differences between a standard laparoscopic procedure and the transvaginal approach by THL. Firstly, in the absence of a panoramic view operative procedures are limited, as we are in close contact with the organs; the latter however allows us to have a clear visualisation of the lesions with increased accuracy due to the use of a watery distension medium. Furthermore, the operative sheet has only one working channel allowing the introduction of only one 5 Fr instrument.

In the group of 71 patients counselled to attempt a spontaneous pregnancy, a pregnancy rate of 73.2% was obtained. One can discuss in this retrospective analysis if this is due to the operative treatment of the endometriotic lesions, but after a mean infertility duration of 26.5 months and a mean age of 31.25 (±2.6) it is unlikely to expect such a high spontaneous pregnancy rate. Dealing with a growing concern of impaired ovarian reserve after surgery, treatment in these early stages and in absence of markers for progression, using an ablative technique with a bipolar 5Fr probe causes a minimal trauma and a lower risk for recurrences.

## Conclusions

Our results confirms that THL allows a minimally invasive inspection of the female pelvis in patients without obvious pelvic pathology. It enables the detection of lesions of peritoneal and ovarian endometriosis in the very early stages. Treatment of these early stages causes minimal trauma and resulted in a non-IVF pregnancy rate of 43.8%. Although scientifically it would be of interest to know the spontaneous evolution of these lesions without treatment, ethically and medically it is not acceptable not to treat lesions once detected. Although this is the largest study evaluating the effect of treatment of early stages of endometriosis by THL, data should be interpreted cautiously, and no firm conclusion can be drawn on the possible positive effect of the ablative procedure of these early stages of endometriosis on pregnancy rates, due to the retrospective character of our data, and further evaluation is needed.
